# Irradiation and bone marrow reconstitution affect the functional Ly49 natural killer cell repertoire in rats

**DOI:** 10.3389/fcell.2015.00034

**Published:** 2015-05-27

**Authors:** Janne M. Nestvold, Bent Rolstad

**Affiliations:** The Immunbiological Laboratory, Department of Anatomy, Institute of Basic Medical Sciences, University of OsloOslo, Norway

**Keywords:** TBI, BMT, NK cells, MHC-I, Ly49 receptors, mesenchymal stromal cells, rats

## Abstract

Total body irradiation (TBI) is part of the preconditioning regimen for allogeneic bone marrow transplantation (alloBMT) and the procedure is associated with treatment-related toxicity and delayed immune reconstitution. Natural killer (NK) cells develop and acquire functional competence in close interaction with stromal bone marrow cells that are considered relatively radioresistant compared to the hematopoietic compartment. We thus undertook a study to assess the effect of TBI on the reconstitution of class I MHC-specific Ly49 NK cell receptors in a rat model of alloBMT. In rats subjected to TBI alone or followed by MHC-matched BMT, the irradiation conditioning induced a skewing of the Ly49 repertoire. Specifically, the activating Ly49s3^bright^ subset exhibited increased frequency and receptor density which correlated with augmented alloreactivity relative to untreated control rats. Our results highlight the plasticity of NK cells and indicate that ionizing radiation (IR) affects the stromal compartment and as a consequence the maturation and functional properties of bone marrow-derived NK cells. These changes lasted throughout the 6 months observation period, showing that irradiation induces long term effects on the generation of the NK cell receptor repertoire.

## Introduction

Total body irradiation (TBI) is commonly used in conditioning regimens prior to allogeneic bone marrow transplantation (alloBMT) in the treatment of a variety of malignant and non-malignant disorders (Wheldon and Barrett, 2001). TBI is given with the aim to eradicate malignant or abnormal cells and provide sufficient immunosuppression to allow engraftment of transferred hematopoietic stem cells (HSCs) (Heinzelmann et al., 2006). AlloBMT is associated with high treatment-related toxicities and may cause severe adverse effects like infections and graft-vs.-host disease (GvHD) (Appelbaum, 2005; Ferrara and Reddy, 2006). An HLA-matched sibling donor is preferred to facilitate engraftment and reduce the risk of complications after transplantation (Beatty et al., 1985).

The effect of TBI on the hematopoietic compartment is well-documented, whereas there is limited knowledge of the effect on bone marrow stromal stem cells that survive doses of radiation detrimental to HSCs (Chen et al., 2006; Li et al., 2007). The intravenously injected hematopoietic stem cells home to the BM cavity early after transplantation. HSCs reside primarily in the bone marrow within a specialized microenvironment, the HSC niche (Wang and Wagers, 2011). Mesenchymal stem/stromal cells (MSCs) are a main component of the HSC niche and support hematopoiesis by releasing soluble factors and via direct cell-cell interactions with hematopoietic stem- and progenitor cells (Mercier et al., 2012). Lymphocytes develop from BM-derived HSCs through a common lymphoid progenitor (CLP) (Luetke-Eversloh et al., 2013). While T-cells are generated in the bone marrow and educated in the thymus, Natural killer (NK) cell education and differentiation, including acquisition of both activating and inhibitory receptors, occur primarily in the bone marrow. The process of receptor acquisition and NK cell education remains poorly understood. The MHC-I-specific NK cell receptors and ligands [killer cell immunoglobulin-like receptors (KIRs) and HLA in humans and the analogous Ly49 receptors and RT1 in rats] are highly polymorphic and inherited independently as a haplotype (Rolstad et al., 2001; Yokoyama and Plougastel, 2003). Functional competence of NK cells requires engagement of a self MHC-I-specific inhibitory receptor, a process termed education or licensing, a mechanism that also acquires tolerance to self (Orr and Lanier, 2010).

NK cells in the periphery expressing inhibitory receptors that can bind “self” MHC-I are considered licensed with cytokine-producing and cytotoxic functions. The “missing-self” hypothesis proposed a principle for NK cell recognition, where the altered or absent expression of MHC-I molecules is sufficient to induce activation. NK cell responses are subjected to the balance of signaling through inhibitory vs. activating receptors (Rolstad et al., 2001; Moretta et al., 2002). In earlier experiments, we observed that rats exposed to whole body gamma irradiation prematurely gray, whether bone marrow transplanted or not. A study in mice suggests that ionizing radiation induces DNA damage and triggers differentiation of MSCs into melanocytes. The premature graying of hair is a physiological consequence of reduction in the pool of remaining MSCs (Inomata et al., 2009). To examine the effect of IR on the MSCs in the BM microenvironment, we monitored the recovery of peripheral NK cell subsets that had been differentiated and educated in an irradiated host and thereby investigated a possible influence of IR on the NK cell development.

We have employed a rat non-myeloablative transplantation protocol involving sublethal TBI and bone marrow as the source of HSCs. The PVG rat expresses an extensive repertoire of both inhibitory and activating Ly49 receptors and was used as model. Here we compare the expression of Ly49 NK cell subsets in a group of rats subjected to TBI followed by MHC-matched BMT with those of untreated negative control animals. To examine the influence of TBI on the mesenchymal vs. the hematopoietic compartment, a group of rats was exposed to irradiation alone, without transfer of HSCs. After 6 months of follow-up, donor-derived NK cells from the different groups were evaluated for cytolytic activity against MHC-disparate target cells in functional NK cell cytotoxicity assays *in vitro* and *in vivo*.

## Materials and methods

### Animals and experimental approval

The panel of MHC-congenic rat strains PVG.7B (*RT1^c^*), PVG.1N (*RT1^n^*) and PVG.1U (*RT1^u^*) on the Piebald Virol Glaxo (PVG) background bred at the Institute of Basic Medical Sciences, University of Oslo, were originally a kind gift from Dr. Geoffrey Butcher, the Babraham Institute, Cambridge. PVG (*RT1^c^*) rats were purchased from Harlan Laboratories. PVG.7B rats (*RT1^c^*, CD45.2,) are identical to PVG (*RT1^c^*, CD45.1) except for the allelic variant of the leukocyte common antigen CD45 that can be detected with the anti-CD45.2 mAb (His41) (Kampinga et al., 1990). PVG.7B rats were used as donors in the transplantation experiments to enable flow cytometric tracking of donor (His41^+^) NK cells, while PVG rats served as recipients. Male rats, 8–12 weeks old and weighing 200–250 g, were randomly divided into experimental groups. The animal studies were approved by the Experimental Animal Board under the Ministry of Agriculture of Norway and conducted under license number 12.1515 in conformity with “the Norwegian Regulations on Animal Experimentation” and “The European Convention for the Protection of Vertebrate Animals used for Experimental and other Scientific Purposes.” The laboratory animal facilities are subject to a routine health monitoring program and the animals were approved free of infectious organisms according to FELASA recommendations.

### Total body irradiation and bone marrow transplantation

Rats subjected to γ-irradiation alone were exposed to a dose of 6 Gy using a ^137^Cs source (Gammacell 3000 Elan, MDS Nordion), which allowed repopulating of hematopoietic cells in the irradiated rats. Recipient rats for BMT were irradiated with 8.5 Gy, which is close to the median lethal dose (LD_50_) in the PVG rat, estimated in an earlier study (Nestvold et al., 2008). Isolation of bone marrow cells (BMCs) was obtained by flushing the tibias and femurs with 20 ml Gibco RPMI 1640 containing 2% FBS (Invitrogen). Cells were passed through a nylon cell-strainer and mononuclear cells purified by density gradient centrifugation on Nycoprep 1.077A (Medinor ASA). BMCs were depleted of T-cells with a combination of the mouse monoclonal antibodies, culture supernatant and ascites fluid, against CD5 (OX19), TCRα/β (R73) and anti-mouse IgG-coated Dynabeads (Invitrogen). Recipient rats were anaesthetized with Hypnorm (fentanyl citrate and fluanisone, VetaPharma Ltd.) at doses of 0.6 ml/kg that provided sufficient degree of sedation and analgesia through the entire transplantation procedure. After TBI, the recipient rats were injected intravenously 1–2 h later with 25 × 10^6^ BMCs. The animals were evaluated daily for clinical signs of transplant-related adverse events throughout the follow-up period of 6 months.

### Flow cytometry and mAbs

Blood samples, 0.3 ml of blood from the lateral tail vein, were collected in 75 mm heparinized tubes (Drummond, Hemato-Clad, Sigma-Aldrich) and lysed via incubation in 0.8% ammonium chloride lysis buffer. Mononuclear cells were stained with monoclonal antibodies against CD3 (G4.18-FITC/PE), NKR-P1A/CD161a (10/78-FITC/PE) purchased from BD Biosciences, CD45.2 (His41-FITC) kindly provided by Dr. Kampinga, University of Groningen, the Netherlands. Anti-NKR-P1B (STOK27-Alexa647) was produced from hybridomas and conjugated according to standard protocols. The biotinylated antibodies against Ly49i2 (STOK2), Ly49s3 (STOK6), Ly49s3,i3,s4,i4 (DAR13), Ly49s5,i5 (Fly5), Ly49 cocktail (STOK2, STOK6, DAR13, Fly5), NKR-P1A (3.2.3) and CD3 (G4.18) were followed by RPE-Cy5-conjugated streptavidin (Dako). During long-term studies, blood samples were pooled from untreated rats at different ages and used as controls. For each analysis, the level of fluorescence intensity was adjusted to samples from the control rats. All flow cytometry data was analyzed on a FACScalibur cytometer equipped with the CellQuest software (BD Biosciences).

### *In vitro* NK cytotoxicity assays

NK cells from spleens of treated and untreated rats were assessed for cytolytic activity in a ^51^Cr-release assay performed in accordance with previously described procedures (Naper et al., 1995). Splenic mononuclear cells were obtained by Lymphoprep (Axis-Shield) density gradient centrifugation.

NK cells for IL-2 activation were isolated from splenocytes by negative selection with Dynabeads (M-450 SaM IgG, Invitrogen) coated with anti-CD3 mAb followed by positive selection with anti-NKR-P1A mAb-coated beads. Purified NK cells were cultured in medium (RPMI 1640, 25 mM Hepes, L-glutamine, 100 U/ml penicillin, 100 μg/ml streptomycin, 5 × 10^−5^M 2-ME, 1 mM sodium pyruvate and 0.1 mM non-essential amino acids and 10% FBS, all from Invitrogen), supplemented with rat recombinant IL-2. In antibody-blocking experiments, 5 μg of purified mAb DAR13 was added to the effector cells 20 min before addition of targets.

Freshly isolated NK cells were purified from splenocytes using the MACS cell separation system (Miltenyi Biotec) to first deplete the mononuclear cell population of CD3^+^ cells and then enrich for NKR-P1A^+^ cells. T-cells were depleted by incubation with biotinylated antibodies against CD5 (OX19) and CD6 (OX52) followed by anti-biotin microbeads and negative MACS selection using an LS column. NK cells were positively selected by anti-NKR-P1A mAb (3.2.3-biotin) in combination with anti-biotin microbeads.

Target cells were ConA-activated lymphoblasts from PVG.1N or the NK-sensitive mouse lymphoma cell line, YAC-1. Target cells (10 × 10^6^ cells ml^−1^) were incubated with 3.7 MBq of Na^51^_2_CrO_4_ ml^−1^ (Amersham) at 37°C for 1hr. ^51^Cr-labeled targets (1 × 10^5^ cells ml^−1^per well) and serial dilutions of effector cells at the indicated E:T ratios, were plated in 100 μl of complete RPMI 1640 in U-bottomed 96-well plates. ^51^Cr-release was measured after incubation for 4 h at 37°C. Supernatants were harvested with a Titertek harvesting system (Skatron) and radioactivity assessed in a gamma counter (Beckmann). Lysis was determined using the formula (experimental cpm – spontaneous cpm) × 100/(maximum cpm - spontaneous cpm). Spontaneous cpm was measured by incubating targets in medium alone and was <15% of total cpm.

### *In vivo* measurement of allogeneic lymphocyte cytotoxicity (ALC)

Determination of ALC was performed as previously detailed (Rolstad et al., 1985; Lövik et al., 2001). In short, mesenteric and cervical lymph node cells from donor rats were filtered through a nylon cell-strainer and 10–15 × 10^6^ lymphocytes per ml were labeled with 0.4 MBq Na^51^_2_CrO_4_ml^−1^.^51^Cr-labeled cells (10–15 × 10^6^ per rat) were injected intravenously and after 24 h recipient rats were terminated and cervical and mesenteric lymph nodes harvested. Radioactivity was assessed using a gamma counter (Beckman), where ALC is defined as the ratio of radioactivity retained per mg lymph node of allogeneic versus syngeneic recipients, i.e., the lymph node (LN) index. Levels of radioactivity are a measurement of the degree of donor lymphocyte eradication, where a lymph node index < 0.5 indicates a strong ALC and a rapid elimination by recipient NK cells.

### Statistical analysis

Statistical significance between test and control groups was evaluated using a non-parametric Wilcoxon two sided rank test or a Wilcoxon-van Elteren test for multiple paired sets of samples. *P*-values at ≤ 0.05 were considered significant.

## Results

### Rapid recovery of NK cells after T-cell depleted bone marrow graft enriched for NK cells

**Figure 1:**
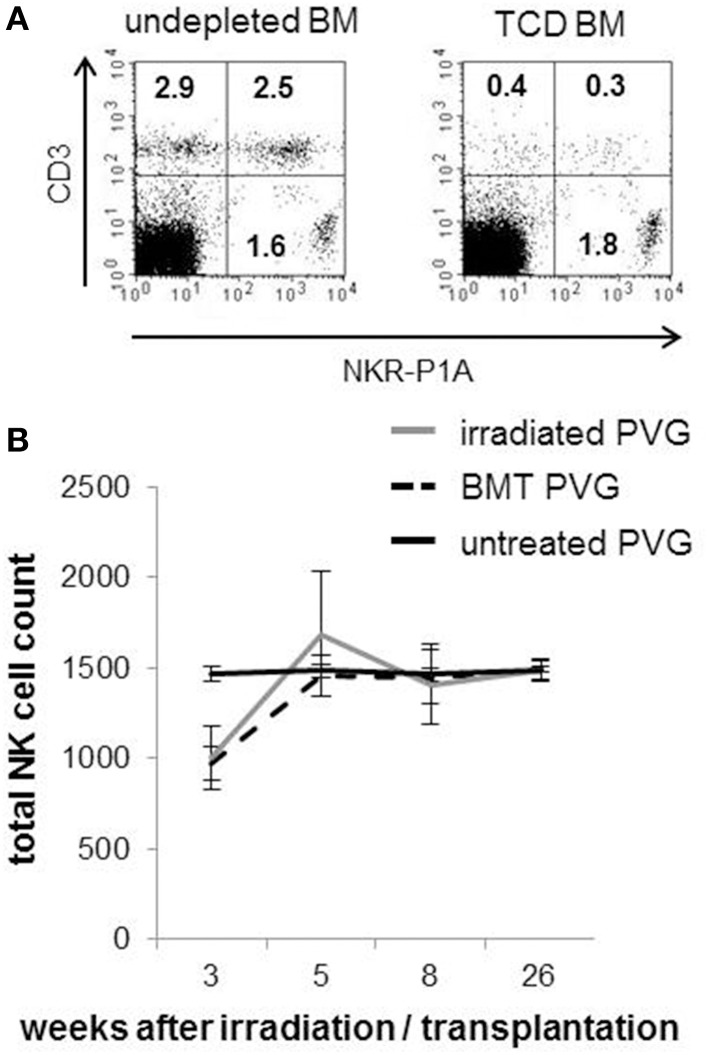
**Rapid recovery of NK cells after MHC-matched BMT**. **(A)** Representative dot plots showing the frequencies of T cells (CD3^+^/NKR-P1A/B^−^, upper left panel), NKT cells (CD3^+^/NKR-P1A/B^+^, upper right panel) and NK cells (CD3^−^/NKR-P1A/B^+^, lower left panel) among the mononuclear bone marrow cells used to reconstitute the rats before (right plot) and after (left plot) depletion of T-cells. **(B)** A standard volume of peripheral venous blood was collected from irradiated PVG, MHC-matched (PVG.7B→PVG) BM-transplanted rats and PVG controls at indicated weeks after treatment and processed for flow cytometry. The kinetics of total NK cell (CD3^−^NKR-P1A^+^ lymphocytes) counts is shown. Values are expressed as average ± SD and data are representative of two independent experiments with six rats per group.

The composition of T- and NK cells in the T-cell depleted (TCD) BM graft used to reconstitute the animals is shown (Figure 1A). The reduction of T-cells after depletion was sufficient to avoid any adverse events. The kinetics of NK cell reconstitution was similar between the irradiated and BMT groups. In line with clinical HSCT (Seggewiss and Einsele, 2010), NK cells were the first lymphocytes to recover and appeared in quantities similar to those found in healthy donors already 5 weeks after transplantation (Figure 1B). The total NK population reflected donor-derived NK cells verified by the use of donor PVG.7B with CD45.2 background in combination with recipient PVG expressing CD45.1.

### Irradiated rats prematurely gray

**Figure 2:**
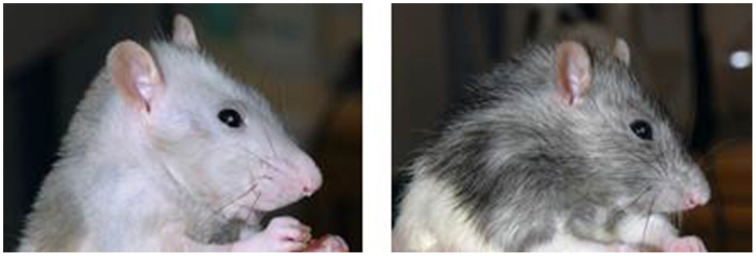
**Irradiated rats prematurely gray**. Depicted is the change in fur color in a rat 1 year after TBI conditioning followed by MHC-matched BMT (photo left side). The right side photo shows an age-matched untreated rat, in which the fur color is unchanged and normal.

A group of irradiated, BM-transplanted and untreated PVG rats were kept for 12 months for monitoring of possible chronic or late effects associated with radiation therapy or BMT. The only macroscopic changes observed were the grayness in fur color accompanied by relative hair loss in treated rats compared to untreated age-matched rats (Figure 2).

### NK cell education after BMT: influence of irradiation on the development of donor-derived NK cells

The prematurely graying of the coats in rats receiving TBI may be a sign of genetoxic stress induced by ionizing radiation (Inomata et al., 2009). This observation led us to investigate the impact and effect of radiation exposure on the development of the NK cell repertoire over an extending period of time after transplantation. Several Ly49 receptors have been characterized in the rat (Naper et al., 2002, 2005, 2010; Kveberg et al., 2006a,b) (Table 1).

**Table 1:** **Monoclonal antibodies against NK cell receptors with strain distribution of their corresponding ligands**.

**mAb**	**Receptor**	**Ligand**	**Produced in**
STOK2	Ly49i2	RT1-A1^c^	DA rats
		Present in PVG	
STOK6	Ly49s3	RT1-CE	DA rats
		High expression in PVG.1N	
		Low expression in PVG	
		Absent in PVG.1U	
DAR13	Ly49s3,i3,s4,i4	RT1-CE	Balb/c mice
		High expression in PVG.1N	
		Low expression in PVG	
		Absent in PVG.1U	
Fly5	Ly49s5,i5	RT1-CE	Balb/c mice
		Absent in PVG	
STOK27	NKR-P1B	Clr11	BN rats
		Present in all haplotypes	

RT1-1A : classical MHC-I.

RT1-CE : non-classical MHC-I.

PVG.1N : MHC complex derived from BN.

PVG.1U : MHC complex derived from AO.

**Figure 3:**
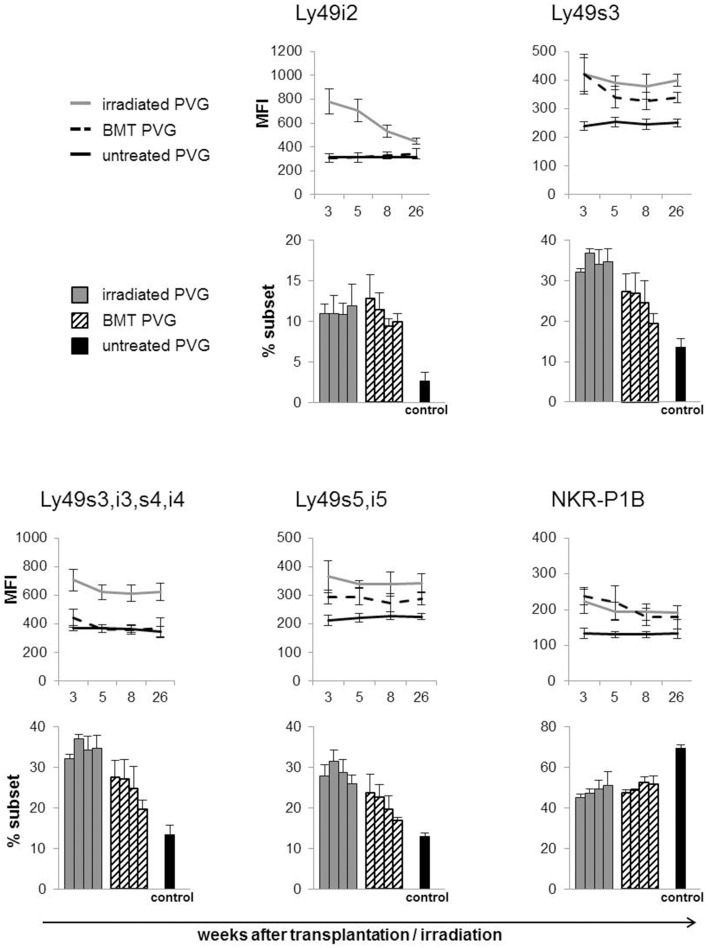
**Total body irradiation induced alterations in the NK cell receptor repertoire**. Peripheral blood samples acquired at denoted weeks from irradiated, BM-transplanted (PVG.7B → PVG) and untreated rats on gated NK cells stained for the indicated receptors. Line diagram upper panel depicting the MFI (average ± SD) of the different Ly49 subsets and the NKR-P1B subset using six rats in each group per time point. Bar diagrams lower panel charting the frequency of the different NK cell subsets for each group of treated and untreated rats. The four parallel columns represent values from the same weeks after treatment as the diagrams with the MFI data, i.e., week 3, 5, 8, and 26 from left to right. Values are expressed as average ± SD. Data are representative of two independent experiments with six rats per group per time point.

**Figure 4:**
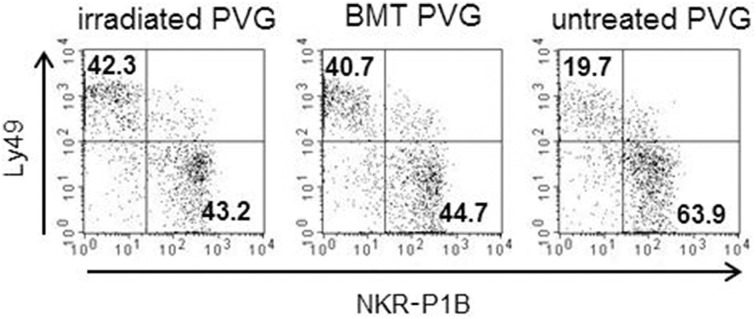
**Irradiation was associated with a skewing toward the Ly49^+^subsets**. Proportion of the alloreactive Ly49^+^ population (upper left quadrant) vs. the non-alloreactive NKR-P1B^+^ population (lower right quadrant) from irradiated, BM-transplanted (PVG.7B → PVG) and untreated controls. Numbers in quadrants show the percentage of cells in each quadrant. Data are representative of three independent experiments.

Subsets of NK cells in peripheral blood were quantified by flow cytometry in terms of frequency of receptor positive cells and by estimation of mean fluorescence intensity (MFI) (Figure 3). The Ly49 mAbs STOK2 and STOK6 interact with an inhibitory and an activating receptor respectively. In MHC-matched BM-transplanted rats the density level of Ly49i2 was similar to untreated rats, while Ly49s3 expression was persistently higher. The mAbs DAR13 and Fly5 cross-react with both activating and inhibitory receptors and although the subsets defined by these antibodies were all increased in proportions their relative contribution is not known or possible to evaluate by flow cytometric analysis. The NKR-P1B receptor, the ligand of which is encoded outside the MHC, exhibited a higher expression level in BM-transplanted and irradiated rats compared to controls. Rats subjected to irradiation alone, showed an increased expression of all the MHC-I-dependent Ly49 receptors. In this scenario, the hematopoietic compartment is exposed to IR and also account for the alteration in the receptor repertoire. In all the treated animals we found an overall expansion of the Ly49 subsets, while the NKR-P1B subset decreased relative to untreated rats, summarized in Figure 4.

### Upregulation in the activating Ly-49s3^bright^ subset in MHC-matched transplanted rats

**Figure 5:**
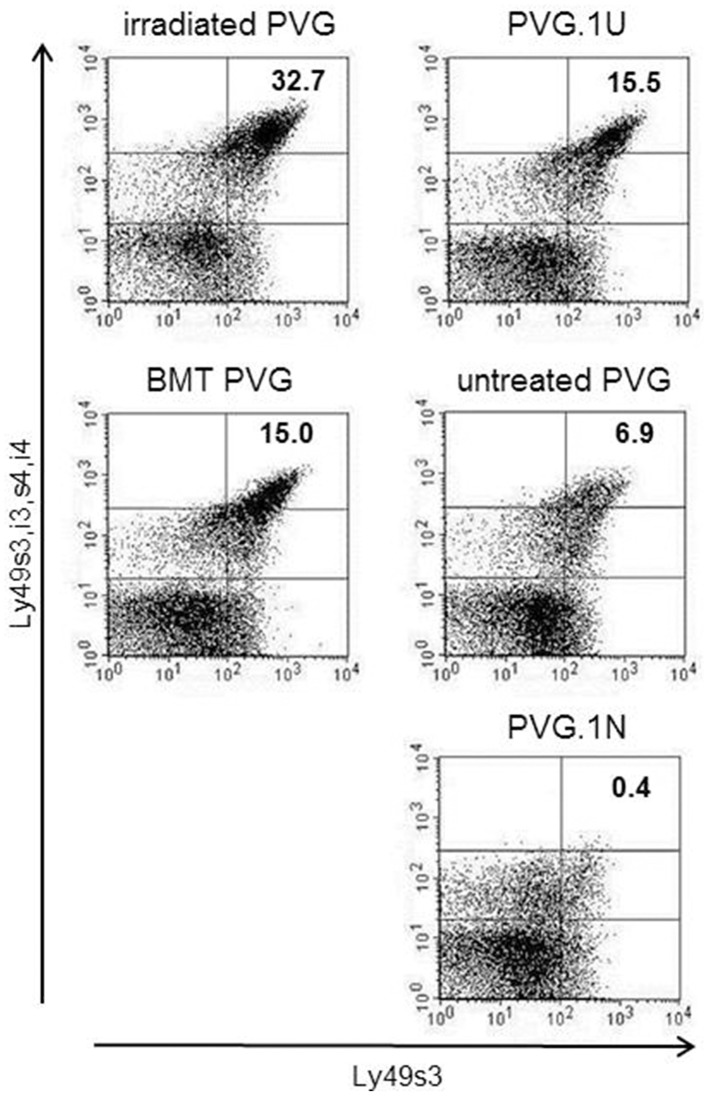
**Upregulation of the activating Ly49s3^**bright**^ subset in irradiated rats**. Representative dot plots from blood samples of the treated groups (left panel), where numbers denoted in plots represent the percentages of NK-gated Ly49s3^bright^ expressing cells (upper right quadrant), compared to untreated control rats (right panel) possessing no ligand expression (PVG.1U) low ligand levels (PVG) or high ligand expression (PVG.1N) for Ly49s3. Data are representative of three independent experiments.

The expression level of Ly49s3 was enhanced in treated rats, determined by labeling with the STOK6 mAb, which is specific but has low affinity for Ly49s3. To assure the identity of the Ly49s3 receptor, we combined the STOK6 mAb with the DAR13 mAb, which expresses high affinity for Ly49s3 as well as Ly49i3,s4,i4. Labeling with these two mAbs using different fluorochromes, revealed a distinct Ly49s3^bright^ population (Figure 5). It was evident that the Ly49s3 receptor showed increased receptor expression in rats with no (PVG.1U) or low-affinity (untreated PVG) ligand and decreased expression in PVG.1N, which exhibits a high-affinity ligand. Results from the control rats demonstrated that NK cells in peripheral blood follow the principle of ligand-dependent receptor expression as shown in earlier murine studies that NK cells developing in the presence of their cognate ligands, express lower levels of the corresponding Ly49 receptors and vice versa. There was a notable increase in the size of the Ly49s3 subset in the MHC-matched transplanted group compared to untreated PVG rats and the increased amount of this subset in the irradiated PVG group, above the level of PVG.1U controls, lacking the cognate ligand.

### Increased frequency of Ly49 subsets in donor-derived NK cells was associated with higher degree of *in vitro* and *in vivo* cytotoxicity

**Figure 6:**
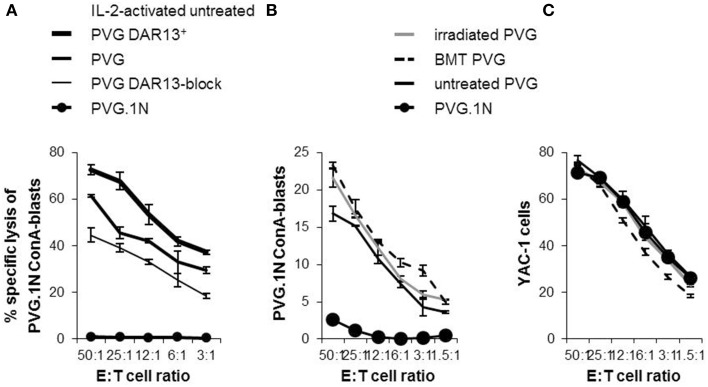
**Expression levels of NK cell Ly49 receptors correlated with the cytolytic capacity**. ^51^Cr -release assays showing the alloreactive potential of IL-2-activated PVG NK cells towards PVG.1N ConA-blasts **(A)**. “PVG DAR13^+^” is subpopulations enriched for Ly49s3,i3,s4,i4 receptors and “PVG DAR13-block” refers to blocking experiment by adding DAR13 mAb to the cytotoxicity assay of the complete NK cell population. Ex vivo isolated splenic NK cells from treated rats at week 26 and untreated controls tested at varying effector: target (E:T) ratios against **(B)** PVG.1N ConA blasts and (**C**) YAC-1 cells. The results are reported as average of cpm ±SD of triplicates from two independent experiments.

In earlier studies using MHC-congenic rat strains, the DAR13^+^ subset in PVG rats is assumed to be mainly responsible for the cytotoxic potential against MHC-mismatched targets (Kveberg et al., 2006b). To evaluate the cytolytic capacity of the DAR13^+^population, we tested Il-2 activated NK cells from PVG against MHC-mismatched PVG.1N lymphoblasts that were efficiently killed (Figure 6A). Most of the reactivity in the first combination was among the DAR13^+^ (Ly49s3,i3,s4,i4) NK cells, since enrichment of this subset increased the cytotoxic response. The effect of DAR13 blockade of the total NK effector cell population was not complete. This may be due to incomplete receptor inhibition or indicating a significant contribution of other forms of stimulatory NK cell recognition, e.g., upregulation of NKG2D ligands on ConA blasts.

To investigate whether the increased proportion of Ly49 receptors and the enhanced levels of the Ly49s3^bright^ receptor in the irradiated rats or the irradiated bone marrow chimeras vs. untreated rats were accompanied by a change in the cytotoxic potential, we performed *in vitro* cytotoxicity assays against the same MHC-mismatched target (PVG.1N). Since IL-2 activated NK cells may show broader cytolytic response not necessarily connected only with allospecificity, we here tested alloreactivity of freshly isolated NK cells (Figure 6B). NK cells from MHC-matched transplanted and irradiated PVG rats killed PVG.1N ConA blasts avidly and slightly more effective than untreated PVG controls (*P* < 0.05). PVG.1N NK cells as controls were non-lytic against syngeneic blasts. NK cells from both treated and untreated rats killed the standard NK tumor target YAC-1 to the same degree (Figure 6C).

Upregulation of the activating Ly49s3^bright^ subset of donor-derived NK cells after MHC-matched BMT may provide an explanation for the increased cytolytic capacity of these NK cells toward PVG.1N blasts *in vitro*. We therefore extended cytotoxicity assessment *in vitro* to an *in vivo* assay for NK alloreactivity termed Allogeneic Lymphocyte Cytotoxicity (ALC) (Rolstad et al., 1985). Here we also employed additional target cells with a different MHC haplotype (PVG.1U). Table 2 shows elimination of transplanted ^51^Cr-labeled PVG.1N or PVG.1U lymphocytes 24 h after iv. transfer in treated and untreated groups of recipients. The ALC lymph node (LN) index in MHC-matched transplanted rats and PVG rats exposed to irradiation alone was lower than for untreated PVG rats demonstrating stronger rejection of MHC disparate targets in treated rats. The difference was significant (*P* < 0.05) in rats injected with PVG.1N lymphocytes, and there was also a tendency to increased elimination after injection of PVG.1U cells although the small numbers did not allow statistical analysis. Thus, the rapid eradication of lymphocytes *in vivo* correlated closely with our *in vitro* findings and indicates that irradiation alone had an enhancing effect on the ability of NK cells to eliminate allogeneic lymphocytes.

**Table 2:** ***In vivo*** **allogeneic cytotoxicity (ALC)**.

**Recipient**	**Donor PVG.1N**		**Donor PVG.1U**	
	***n***	**LN index**	***n***	**LN index**
Irradiated PVG	6	0.12 ± 0.03	2	0.05 (0.04–0.05)
BMT (PVG.7B → PVG)	6	0.13 ± 0.03	2	0.05 (0.05)
untreated PVG	6	0.25 ± 0.04	2	0.12 (0.11–0.12)

PVG.1N : MHC complex derived from BN.

PVG.1U : MHC complex derived from AO.

In vivo cytolytic activity of donor-derived NK cells at week 26 in treated groups compared to untreated controls (PVG). A lymph node (LN) index < 0.5 indicates rejection of the inoculated radiolabeled MHC-disparate lymphocytes. Numbers are average ± SD for donor PVG.1N recipients with six rats per group. Ranges of values are given for donor PVG.1U recipients with two rats per group. Data are pooled from two independent experiments.

## Discussion

One salient feature of both the Ly49 expression and the NK alloreactivity data was that irradiated PVG rats and MHC-matched transplanted rats displayed a higher proportion of Ly49^+^ NK cells and an upregulation of the Ly49s3^bright^ subset, which in turn led to augmented NK alloreactivity *in vivo* compared to untreated PVG rats. Long term studies showed altered expression frequencies and levels of these receptors that were stable for several months and not a transient phenomenon connected to recovery of the NK cell population early after irradiation and BMT. These persistent alterations suggest that IR may lead to permanently changes in receptor expression of donor-derived NK cells. The results indicate that development of the NK cell repertoire after TBI does not precisely mimic normal ontogeny of NK cells.

The bone marrow is considered the main site of NK cell development, although extramedullary sites also can support NK cell differentiation (Luetke-Eversloh et al., 2013; Yu et al., 2013). MSCs do not engraft after BMT and stroma remains to be of host origin (Dickhut et al., 2005; Rieger et al., 2005). Radiosensitive HSCs undergo apoptosis in response to low doses of IR exposure whereas MSCs are characterized as more radioresistant (Sugrue et al., 2013). Ionizing radiation may directly produce DNA double strand breaks (DSBs) and is also related to an increase in cellular stress and formation of reactive oxygen species (ROS) (Hoeijmakers, 2009). ROS induce oxidative cellular damage and are involved in mediation of acute and chronic inflammatory responses. MSCs reside in BM niches and the hypoxic conditions provide a radioprotective environment. MSCs are shown to possess a robust antioxidant system for ROS detoxification and an effective DNA damage response (DDR) contribute to the radioresistance of MSCs (Sokolov and Neumann, 2013). Preconditioning with TBI may not only rapidly alter soluble and insoluble components of the microenvironment, but also induce chronic effects by inflammation and oxidative stress. We hypothesize that niche composition may change under these conditions and convert “self” environment to “altered self,” explaining the changes in Ly49 receptor expression after TBI.

The relevance of non-targeted biological responses of ionizing radiation therapy (RT) was first discovered and described by Mole (1953). Multiple case studies have documented radiation-induced effects that are designated as bystander effects (in adjacent unirradiated cells), abscopal effects (in unirradiated tissues distant to the irradiated area), and cohort effects (between irradiated cells within the irradiated field) (Blyth and Sykes, 2011). The responses involve intercellular communication and soluble factors released into the local environment and the biological changes may have both harmful and potentially beneficial consequences. However, the precise mechanisms and pathways responsible for these phenomena have not yet been fully elucidated.

NK cell tolerance is established in the bone marrow, whereas T-cells undergo maturation and tolerance induction in the thymus. The effect of radiation exposure on thymus-dependent T-cell reconstitution is incompletely understood. Thymus dysfunction after irradiation preconditioning is related to functional impairment of the thymic stromal cells. The modifications in the stromal compartment may influence maturation, TCR repertoire selection and education to self-tolerant T-cells (Chung et al., 2001; Kelly et al., 2008). Although poorly investigated, ionizing radiation may as well affect the induction of tolerance in developing NK cells by altering the marrow stromal microenvironment.

The effect of TBI on the NK cell development was associated with expansion of the Ly49 repertoire. All Ly49 receptors identified with our antibodies increased in frequency, whether they were exposed to their cognate MHC-I ligand in PVG (Ly49i2 and Ly49s3,i3,s4,i4) or the ligand was absent (Ly49s5,i5). Assumedly, this expansion could be ligand-dependent, e.g., MHC-I or stress-induced ligands altered by TBI or ligand-independent, e.g., cytokine driven response to irradiation. Most notably, in the BM-transplanted rats the density level of the activating Ly49s3^bright^ receptor was significantly increased after TBI whereas the receptor expression of the inhibitory Ly49i2 was unchanged compared to untreated control rats. The process of receptor expression during development of NK cells requires *trans* interactions with MHC-I on hematopoietic cells and stromal cells. In the PVG rat, the Ly49i2 receptor is the main inhibitory receptor and the alloreactivity appears confined to the activating Ly49s3 subset (Naper et al., 1998, 2002). The difference in receptor expression levels of the Ly49s3 receptor between donor-derived NK cells in transplanted rats and NK cells in non-irradiated controls, indicates that IR creates conditions in the BM microenvironment leading to upregulation of activating Ly49 receptors in favor of responsiveness. Along these lines our data suggest a relation to the stromal compartment and presumably by altered expression of activating ligands as a consequence of irradiation.

We aimed to correlate the preconditioning irradiation with a change in the Ly49 repertoire post-transplant along with augmented Ly49-mediated cytotoxicity. While the recovery of adaptive immunity is delayed, NK cells rapidly recover after BMT and may be important in the early phase of immune reconstitution. It is intriguing to speculate, that irradiation conditioning may serve as a useful adjuvant to maximally alert viral resistance and anti-tumor effects of NK cells expressing activating Ly49 receptors (Ruggeri et al., 2002). Susceptibility to cytomegalovirus (CMV) reactivation after HSCT is associated with immunodeficiency in the host early post-transplant, induced by the conditioning regimen. Several MHC-I-dependent activating NK cell receptors have been shown to recognize CMV and may play an important role in the first line defense against infections following HSCT (Orr et al., 2010; Marras et al., 2011).

The prerequisite for therapeutic utilization of NK cells is a better understanding of the NK cell developmental pathways following preconditioning regimens and hematopoietic stem cell transplantation. The knowledge can further be used for improve the efficacy of HSCT and to design specific NK cell-based immune therapies.

## Author contributions

BR, JN designed and performed experiments, analyzed and interpreted data, and participated in the article preparation.

### Conflict of interest statement

The authors declare that the research was conducted in the absence of any commercial or financial relationships that could be construed as a potential conflict of interest.
